# Educational attainment inequalities in depressive symptoms in more than 100,000 individuals in Europe

**DOI:** 10.1192/j.eurpsy.2020.100

**Published:** 2020-11-16

**Authors:** Adam Chlapecka, Anna Kagstrom, Pavla Cermakova

**Affiliations:** 1 Third Faculty of Medicine, Charles University Prague, Prague, Czech Republic; 2 National Institute of Mental Health, Klecany, Czech Republic; 3 Second Faculty of Medicine, Charles University Prague, Prague, Czech Republic

**Keywords:** Depression, education, epidemiology, Europe

## Abstract

**Background:**

Increasing educational attainment (EA) could decrease the occurrence of depression. We investigated the relationship between EA and depressive symptoms in older individuals across four European regions.

**Methods:**

We studied 108,315 Europeans (54% women, median age 63 years old) from the Survey on Health, Ageing and Retirement in Europe assessing EA (seven educational levels based on International Standard Classification of Education [ISCED] classification) and depressive symptoms (≥4 points on EURO-D scale). Logistic regression estimated the association between EA and depressive symptoms, adjusting for sociodemographic and health-related factors, testing for sex/age/region and education interactions.

**Results:**

Higher EA was associated with lower odds of depressive symptoms, independent of sociodemographic and health-related factors. A threshold of the lowest odds of depressive symptoms was detected at the first stage of tertiary education (OR 0.60; 95% confidence interval [CI] 0.55–0.65; *p* < 0.001; relative to no education). Central and Eastern Europe showed the strongest association (OR for high vs. low education 0.37; 95% CI 0.33–0.40; *p* < 0.001) and Scandinavia the weakest (OR for high vs. low education 0.69; 95% CI 0.60–0.80; *p* < 0.001). The association was strongest among younger individuals. There was a sex and education interaction only within Central and Eastern Europe.

**Conclusions:**

Level of EA is reflected in later-life depressive symptoms, suggesting that supporting individuals in achieving EA, and considering those with lower EA at increased risk for depression, could lead to decreased burden of depression across the life course. Further educational support in Central and Eastern Europe may decrease the higher burden of depressive symptoms in women.

## Introduction

The World Health Organization ranked depression as the third largest burden of disease globally, expecting it to rank first by 2030 [1]. Risks for depression include social, economic, geographic, and biological factors, which are reflected through inequitable distribution of depression across European subpopulations, with the highest burden in women, less educated individuals with fewer socioeconomic resources, and within Southern and Central and Eastern Europe (CEE) [2–5]. These discrepancies indicate that reduction of these inequalities could result in diminished risk for depression in Europe. Several early life risk factors, including limited access to education, make significant contributions to the development of depression across the life course [6].

Educational attainment (EA) is one of the most prominent social determinants of health as evidenced by decades of research [7,8]. Along with depression [9], lower EA is associated with decreased life expectancy [10] and increased risk of dementia [11], disability [12], cancer [13], and heart disease and diabetes [14]. EA has more recently been identified as a strong predictor of adult health and longevity, particularly within the US [9,15,16] and several European countries [17,18]. It is widely discussed whether the association between EA and depression is causal or explained by other sociodemographic or health-related factors [19–21]. EA has been theorized as having effect on health outcomes, since education affords individuals greater access to resources such as fulfilling jobs, economic security, social ties, a sense of personal control, and learnt effectiveness [8]. These resources provide individuals with higher socioeconomic status and improved access to health care and a healthy life style, ultimately leading to improved health.

While EA is an established important health indicator, particularly for somatic illness, the relationship between EA and mental illness is less understood across the life course. Higher EA may armor an individual with qualities, such as ability to pursue meaning in life, sense of proficiency, and better ability to cope with stressful events, which may all provide protection against depressive symptoms, irrespective of somatic diseases and socioeconomic status. The relationship between EA and depressive symptoms is considered bidirectional [4,19,22]. Lower EA can lead to social risks, for example, lower socioeconomic status, less effective coping strategies to mitigate stressors, and less healthy lifestyle, leading to compounded risk for depression [4,19,22]. Predisposition for depression is also thought to lead to lower EA, for example, when the onset of symptoms is experienced prior to the completion of educational goals of respective individuals. As our cohort consists of older adults, we focus primarily on the association with EA on later-life depression, given that education is typically undertaken during childhood and/or young adulthood.

The majority of past studies, analyzing a potentially protective effect of EA on depression, have assumed few boundaries to the mental health benefits of EA, supported by evidence of far-reaching benefits of EA spanning physical, social, relational, and individual dimensions [8]. This would suggest that there is no upper threshold to the mental health benefits of EA. However, some studies suggest that the benefits of EA on mental health are not proportional and diminish for those with high EA [8,23]. Large representative studies on the association between EA and depression are limited. Our study aimed to (a) examine whether higher EA is associated with lower depressive symptoms, when sociodemographic and health-related factors are accounted for, (b) determine, if there is an upper limit to this association, beyond which no extra benefits of EA can be observed, and (c) explore regional and demographic differences within the relationship between EA and depressive symptoms across Europe.

## Methods

### Participants

The analysis is based on data from Survey on Health, Ageing and Retirement in Europe (SHARE), as previously described [24]. Briefly, data are collected in individuals aged at least 50 years and their partners, using a computer-assisted personal interview (CAPI). The first wave was conducted in 2004 and was followed by six subsequent waves. SHARE has been repeatedly reviewed and approved by the Ethics Committee of the University of Mannheim. All participants signed an informed consent.

### Educational attainment

Information about EA was collected as a part of CAPI in the same wave when depressive symptoms were assessed and were categorized into seven EA levels based on the International Standard Classification of Education (ISCED) - 97 [25]: no or pre-primary level of education (level 0), primary level of education (level 1), lower secondary level (level 2), upper secondary level (level 3), post-secondary non-tertiary education (level 4), first stage of tertiary education (level 5), and second stage of tertiary education (level 6). Individuals with the lowest EA levels were the oldest and to the greatest extent women (Supplemental Table S1).

### Depressive symptoms

Depressive symptoms were measured using EURO-D scale [26], which consists of 12 items (depressed mood, pessimism, wishing death, guilt, sleep, interest, irritability, appetite, fatigue, concentration, enjoyment, and tearfulness), scored as 0 (symptom not present) or 1 (symptom present). As previously [2,27], we used the cut-off of 4 or more points indicating the presence of significant depressive symptoms, referred to hereafter as “depressive symptoms.” To test the robustness of this approach, we performed a sensitivity analysis with a cut-off of 7 points (95th percentile), capturing people with the most severe depressive symptoms.

### Covariates

Sociodemographic and health-related covariates were identified based on literature [2–4,27] and were assessed in the wave when depressive symptoms were measured. If there were missing data on a particular covariate in that wave, the value from the closest wave was used. After this procedure, each covariate included <5% of missing data. Descriptive analysis was conducted on the sample that included also individuals with missing data, while multivariable analysis was performed on complete cases. Sociodemographic characteristics were age (years), sex (women vs. men), household net worth (standardized difference between household gross financial assets and financial liabilities), employment status (working vs. not working), family status (living with a partner vs. alone), and number of children and grandchildren.

Health-related characteristics were number of limitations in instrumental activities of daily living (IADL), cardiovascular disease, cognitive status based on 10 words delayed recall test, number of chronic diseases (measured by self-reported physicians’ diagnosis; including heart disease, stroke, hypertension, diabetes or high blood sugar, cancer, lung disease, and general disability [8]), body mass index (BMI), physical inactivity (never vigorous nor moderate physical activity vs. physical activity), smoking (ever smoked daily vs. never smoked daily), alcohol use (drinking more than two glasses of alcohol per day vs. drinking less; one drink is: 1 bottle/can of beer = 33 cl, 1 glass table wine = 12 cl, 1 glass fortified wine = 8 cl, and 1 glass spirits = 4 cl [8]), drugs against depression or anxiety (self-reported and assessed by a question, whether participants use drugs against depression or anxiety [8]), and maximal grip strength.

### Study sample

We studied individuals at the first time they had available data on EURO-D. From the 139,556 individuals who completed at least one interview in SHARE, we excluded those with missing data on depressive symptoms (*n* = 22,247) and EA (*n* = 1,299), individuals younger than 50 years (*n* = 4,233) and participants from Israel (*n* = 3,462), as this study focused on older European population, leaving the sample of 108,315 persons from four European regions: Western Europe (*n* = 44,094), CEE (*n* = 28,611), Southern Europe (*n* = 23,944), and Scandinavia (*n* = 11,666), see Supplemental Figure S1.

### Statistical analysis

Participants´ characteristics are presented as frequency (*n*, %), mean ± standard deviation (SD), or median and interquartile range (IQR). We compared characteristics between individuals with and without depressive symptoms using independent samples *t*-test, Mann–Whitney test, and chi-square test. Subsequently, we employed logistic regression to estimate odds ratio (OR) with 95% CI for the association of EA (with level 0 as reference) with depressive symptoms, adjusting for covariates in three steps. Model 1 included sex and age; Model 2 included sex, age, and remaining sociodemographic characteristics, and Model 3 all sociodemographic and health-related characteristics.

As there are great differences in both EA [28,29] and the burden of depressive symptoms across European regions [2], we explored whether the association between EA and depressive symptoms differs regionally. We first included a two-way interaction term into Model 1 between EA and region and assessed the interaction effect with a likelihood ratio (LR) test. As there was not a sufficient number of participants for each EA in each region, we combined the original seven levels of EA into three groups: low (levels 0 and 1), middle (levels 2–4), and high education (levels 5 and 6). We fit Models 1–3, as described above.

Last, we tested whether sex and age (young than 65 years vs. older than 65 years) act as effect modifiers in the association of EA with depressive symptoms. We included an interaction term between sex/age group and EA into Model 1 in the whole analytical sample as well as in each region. We used LR test to assess the effect of interaction and conducted stratified analyses in case of significant interactions. The analysis was conducted using R software (RStudio Version 1.2.1335).

## Results

**Figure 1.**
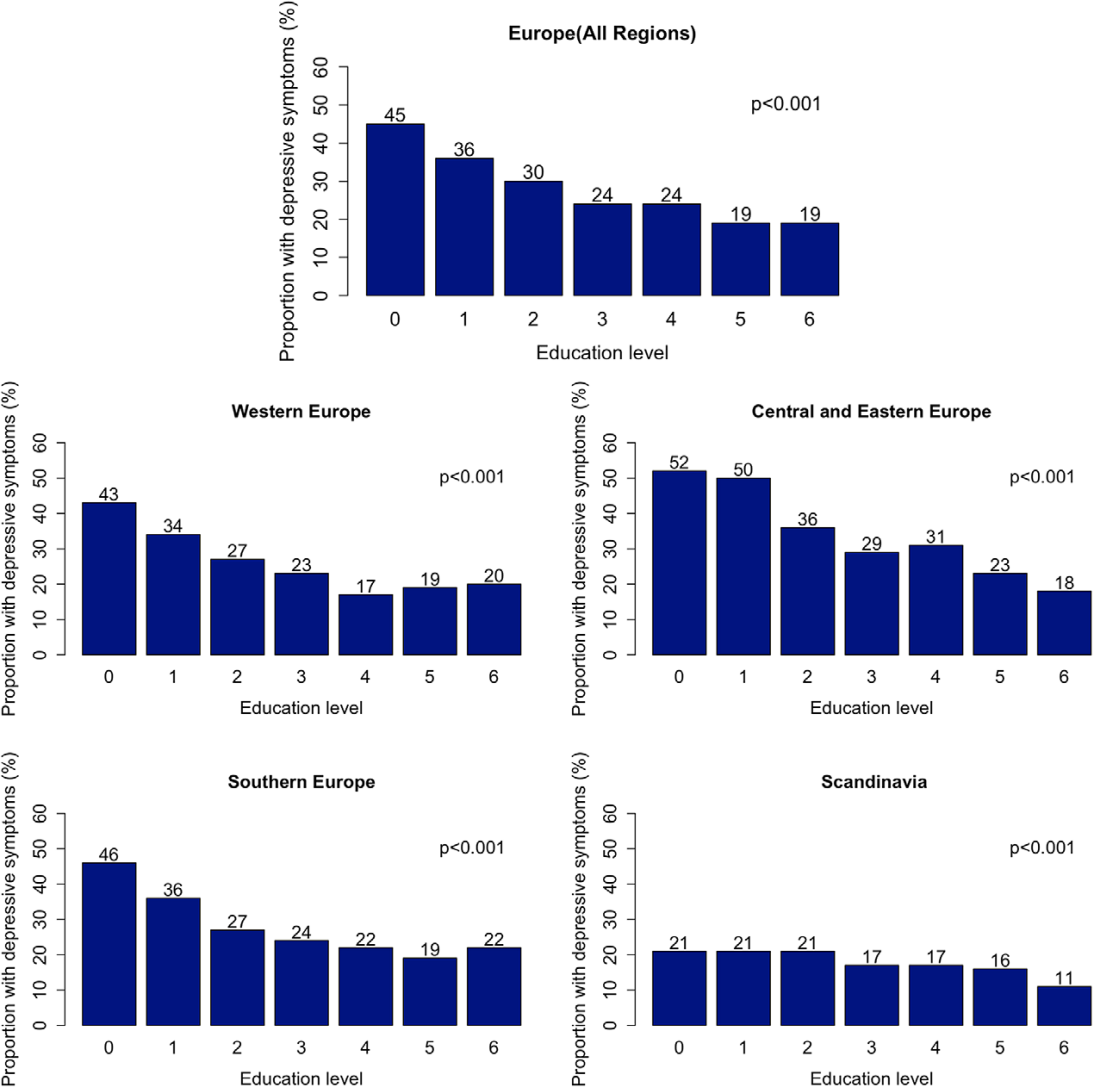
Prevalence of depressive symptoms in different educational attainment levels in Europe and stratified by European region.

Out of 108,315 individuals (median age 63 years; 54% women), 28% (*n* = 29,919) had depressive symptoms (Table 1). Individuals with depressive symptoms were older, more often women, socioeconomically worse off and had a worse health profile. The highest prevalence of depressive symptoms was in the EA level 0 (45%), while the lowest in levels 5 and 6 (both 19%; Figure 1). A lower frequency of depressive symptoms co-occurred with increasing EA but plateaued at level 5. When stratified by region, depressive symptoms were most frequent in EA level 0 across all regions (highest in CEE: 52%, lowest in Scandinavia: 21%). The lowest prevalence of depressive symptoms was in level 6 in CEE (18%) and Scandinavia (11%), but in level 4 in Western Europe (17%) and in level 5 in Southern Europe (19%).

**Table 1.** Characteristics of participants.

	Depressive symptoms (*n* = 29,919)	No depressive symptoms (*n* = 78,396)
*Sociodemographic factors*		
Age, median (IQR)**	64.00 (18)	62.00 (14)
Women, *n* (%)**	20,249 (68%)	38,744 (49%)
Highest decile of household net worth, *n* (%)	2,066 (7%)	8,767 (11%)
Currently working, *n* (%)**	6,354 (22%)	26,797 (34%)
Living with a partner, *n* (%)**	18,788 (63%)	58,889 (75%)
Children, one and more, *n* (%)**	26,695 (89%)	70,666 (90%)
Grandchildren, one and more, *n* (%)**	19,917 (67%)	47,937 (61%)
*Health-related factors*		
At least one limitation in IADL, *n* (%)**	9,661 (32%)	7,482 (10%)
Cardiovascular disease, *n* (%)**	20,488 (68%)	42,407 (54%)
Delayed recall, median words (IQR)**	3.00 (4.00)	4.00 (2.00)
Number of chronic diseases, median (IQR)**	2.00 (2.00)	1.00 (2.00)
BMI, mean ± SD**	27.13 ± 5.16	26.68 ± 4.35
Physical activity, *n* (%)**	23,636 (79%)	72,981 (93%)
Never smoked daily, *n* (%)**	13,819 (46%)	33,740 (43%)
No excessive drinking, *n* (%)**	26,065 (87%)	65,178 (83%)
Drugs for anxiety/depression, *n* (%)**	4,325 (14%)	2,238 (3%)

Abbreviations: BMI, body mass index; IADL, instrumental activities of daily living; IQR, interquartile range.

Note: *p*-values were derived from independent samples *t*-tests, Mann–Whitney *U* tests, and chi-square tests.

*p < 0.05.

** *p* < 0.001.

In the whole sample, adjustments for covariates reduced the magnitude of all associations, but they remained statistically significant (Table 2). When compared to EA level 0, all higher levels were associated with lower odds of depressive symptoms in the model, which adjusted for all covariates (Table 2, Model 3). The magnitude of this association showed incremental gains from level 1 (OR 0.79; 95% CI 0.73–0.85; *p* < 0.001) through level 5 (OR 0.60; 95% CI 0.56–0.66; *p* < 0.001) but decreased at level 6 (OR 0.76; 95% CI 0.62–0.92; *p* < 0.001). Level 5 was a threshold for the observed benefits of the lowest odds of depressive symptoms in all models.

**Table 2.** Association of educational attainment with depressive symptoms.

EA	Model 1	Model 2	Model 3
Level 0	Reference	Reference	Reference
Level 1	0.69 (0.65; 0.74)**	0.68 (0.64; 0.73)**	0.79 (0.73; 0.85)**
Level 2	0.55 (0.51; 0.59)**	0.55 (0.51; 0.59)**	0.71 (0.66; 0.77)**
Level 3	0.45 (0.42; 0.48)**	0.47 (0.44; 0.50)**	0.67 (0.62; 0.72)**
Level 4	0.42 (0.39; 0.47)**	0.46 (0.42; 0.50)**	0.68 (0.61; 0.75)**
Level 5	0.34 (0.32; 0.37)**	0.39 (0.36; 0.41)**	0.60 (0.56; 0.66)**
Level 6	0.37 (0.30; 0.44)**	0.44 (0.37; 0.53)**	0.76 (0.62; 0.92)*
AIC	122,316	119,886	101,877

Abbreviations: AIC, Akaike information criterion; EA, educational attainment.

Note: Results are odds ratio with 95% confidence intervals derived from logistic regression for the association of EA with depressive symptoms. Model 1: age, sex. Model 2: age, sex, household net worth, employment status, family status, number of children, number of grandchildren. Model 3: age, sex, household net worth, employment status, family status, number of children, number of grandchildren, limitations in instrumental activities of daily living, 10 words delayed recall test, number of chronic diseases, chronic vascular disease, body mass index, physical activity, smoking, excessive alcohol intake, drugs for anxiety/depression, maximal grip strength.

* *p* < 0.05.

** *p* < 0.001.

There was a significant interaction (*p* value from LR test <0.001) between EA and region. When stratified, both middle and high education were associated with lower odds of depressive symptoms in all regions in age-sex adjusted models, when compared to low education (Table 3, Model 1). The magnitude of this association was the greatest in CEE and the smallest in Scandinavia. In the fully adjusted model, the magnitude of the association was still the greatest in CEE (OR for high vs. low education 0.69 95% CI 0.61–0.77; *p* < 0.001) and the association in Scandinavia even lost statistical significance, and the OR became close to unity (OR for high vs. low education 1.00 95% CI 0.85–1.18; *p* > 0.05).

**Table 3.** Association of educational attainment with depressive symptoms stratified by European region.

	Model 1	Model 2	Model 3
Low education	Reference	Reference	Reference
*Western Europe*			
Middle education	0.59 (0.56; 0.63)**	0.63 (0.60; 0.67)**	0.75 (0.71; 0.81)**
High education	0.49 (0.46; 0.52)**	0.55 (0.51; 0.59)**	0.70 (0.65; 0.76)**
Southern Europe			
Middle education	0.69 (0.64; 0.73)**	0.73 (0.68; 0.78)**	0.89 (0.83; 0.96)*
High education	0.51 (0.45; 0.56)**	0.56 (0.51; 0.63)**	0.73 (0.65; 0.83)**
*Central and Eastern Europe*			
Middle education	0.56 (0.52; 0.60)**	0.59 (0.54; 0.63)**	0.75 (0.69; 0.82)**
High education	0.37 (0.33; 0.40)**	0.42 (0.38; 0.46)**	0.69 (0.61; 0.77)**
*Scandinavia*			
Middle education	0.87 (0.77; 0.99)*	0.93 (0.82; 1.07)	1.04 (0.90; 1.21)
High education	0.69 (0.60; 0.80)**	0.82 (0.71; 0.96)*	1.00 (0.85; 1.18)

Abbreviation: EA, educational attainment.

Note: Results are odds ratio with 95% confidence intervals derived from logistic regression for the association of educational attainment with depressive symptoms. Model 1: age, sex. Model 2: age, sex, household net worth, employment status, family status, number of children, number of grandchildren. Model 3: age, sex, household net worth, employment status, family status, number of children, number of grandchildren, limitations in instrumental activities of daily living, 10 words delayed recall test, number of chronic diseases, chronic vascular disease, body mass index, physical activity, smoking, excessive alcohol intake, drugs for anxiety/depression, maximal grip strength.

* *p* < 0.05.

** *p* < 0.001.

The interaction between sex and EA was significant only for CEE (*p* from LR test 0.04) but not for the whole sample (*p* = 0.26) and other regions (Southern Europe *p* = 0.46; Western Europe *p* = 0.30; and Scandinavia *p* = 0.77). In CEE (Table 4), middle and high education were strongly associated with lower odds of depressive symptoms in men, but there was no association of high education with depressive symptoms in women. Surprisingly, middle education was associated with higher odds of depressive symptoms in women in Models 1 and 2, but this association lost statistical significance and became close to unity in Model 3.

**Table 4.** Association of educational attainment with depressive symptoms in Central and Eastern Europe stratified by sex.

	Men	Women
Low education	Reference	
*Model 1*		
Middle education	0.49 (0.43–0.56)**	1.22 (1.04–1.42)*
High education	0.34 (0.28–0.39)**	1.13 (0.92–1.39)
*Model 2*		
Middle education	0.51 (0.45–0.58)**	1.22 (1.05–1.43)*
High education	0.38 (0.32–0.45)**	1.15 (0.94–1.41)
*Model 3*		
Middle education	0.70 (0.61–0.82)**	1.09 (0.91–1.30)
High education	0.64 (0.53–0.78)**	1.06 (0.84–1.32)

Results are odds ratio with 95% confidence intervals derived from logistic regression for the association of educational attainment with depressive symptoms. Model 1: age, sex. Model 2: age, sex, household net worth, employment status, family status, number of children, number of grandchildren. Model 3: age, sex, household net worth, employment status, family status, number of children, number of grandchildren, limitations in instrumental activities of daily living, 10 words delayed recall test, number of chronic diseases, chronic vascular disease, body mass index, physical activity, smoking, excessive alcohol intake, drugs for anxiety/depression, maximal grip strength.

* *p* < 0.05.

** *p* < 0.001.

The interaction between age group and education was significant in the whole sample (*p* < 0.001) but not in any European region (Southern Europe *p* = 0.16; Western Europe *p* = 0.75; Scandinavia *p* = 0.15; and CEE *p* = 0.54). When stratified (Table 5), the magnitude of the association was greater in the younger age group when compared to the older age group in all models. Sensitivity analysis with a higher cut-off for depressive symptoms did not change the results to a great extent (Supplemental Table S1).

**Table 5.** Association of educational attainment with depressive symptoms in the whole analytical sample stratified by age group.

	Age 50–64 years	Age 65 years or older
Low education	Reference	
*Model 1*		
Middle education	0.67 (0.64–0.71)**	0.90 (0.84–0.96)*
High education	0.48 (0.45–0.51)**	0.90 (0.82–0.98)*
*Model 2*		
Middle education	0.74 (0.70–0.78)**	0.85 (0.80–0.91)**
High education	0.60 (0.56–0.64)**	0.79 (0.72–0.87)**
*Model 3*		
Middle education	0.89 (0.84–0.94)**	0.92 (0.85–0.99)*
High education	0.78 (0.73–0.83)**	0.93 (0.84–1.02)

Results are odds ratio with 95% confidence intervals derived from logistic regression for the association of educational attainment with depressive symptoms. Model 1: age, sex. Model 2: age, sex, household net worth, employment status, family status, number of children, number of grandchildren. Model 3: age, sex, household net worth, employment status, family status, number of children, number of grandchildren, limitations in instrumental activities of daily living, 10 words delayed recall test, number of chronic diseases, chronic vascular disease, body mass index, physical activity, smoking, excessive alcohol intake, drugs for anxiety/depression, maximal grip strength.

* *p* < 0.05.

** *p* < 0.001.

## Discussion

In this large population-based cohort study of more than 100,000 individuals residing in 20 diverse countries across four European regions, we observed a lower burden of depressive symptoms associated with higher EA; however, the first stage of tertiary education was a threshold for the benefits of EA, with no apparent protective effect against depressive symptoms observed beyond this level. The relationship between higher EA and lower depressive symptoms was apparent particularly among younger individuals, strongest among individuals in CEE, and weakest in Scandinavia. Differences between men and women were present only in CEE, where middle and high EA were strongly associated with lower odds of depressive symptoms in men; however, high EA was not related to depressive symptoms in women.

Our results are consistent with studies on middle-aged population and twins in the US showing that higher EA may serve as a protective factor against depressive symptoms [30–32] but are not in accord with a twin study by Fujivawara and Kawachi [33] that found EA unrelated to depressive symptoms. In another study examining a UK schooling reform in 1972, which raised the minimum school leaving age from 15 to 16 years old, Avendano et al. found that although the reform increased EA, it did not decrease depression in adulthood [34]. Our study is unique due to the following reasons: First, it includes by far the largest sample; second, its results can be generalized to a wide geographical area including countries from CEE that have been underrepresented in previous research on mental health [35–38]; third, we were able to adjust for a wealth of sociodemographic and health-related characteristics.

Previous studies suggest several reasons why higher EA may be beneficial for mental health. Some propose that the protective effect of higher EA on mental health is explained by factors related to better socioeconomic position and less somatic morbidity [4,19,22]. These findings are in contrast to Bjelland et al. [20] who argued that there is also a direct beneficial effect of EA on mental health. Our study suggests that the association of EA and depressive symptoms is independent of a wide range of sociodemographic and health-related characteristics. Building on this evidence base, we found that the threshold for the EA gradient of depressive symptoms lies at the first stage of tertiary EA in Europe. This is in accord with a US study by Mirowsky and Ross [8] who found fewer benefits related to well-being among individuals with EA beyond a master’s degree level. Reduced mental health benefits in populations with high EA can be also seen in results from Link et al. [39].

The plateau of protective benefits of EA on later-life depression may reflect the value of education as a catalyst for additional protective factors, such as socioeconomic status and health promoting behaviors, which appear to have a ceiling effect. The mental health needs EA can satisfy, such as cognitive and coping skills or learnt effectiveness, can be saturated at the completion of the first stage of tertiary education. In addition, although our models include multiple sociodemographic and health-related factors, there could still be residual differences between participants in the first and second stage of tertiary EA, unaccounted for, such as work satisfaction. The phenomenon of overeducation could explain the threshold found in our cohort, which is traditionally conceptualized as a job–education mismatch, where the level of EA acquired exceeds the level of EA required to adequately perform a job [40]. Bracke et al. [41] suggest that well-educated individuals are at risk of being overqualified for the job, which is associated with mental health complaints.

Our study further indicates that the strongest protective effect of EA on depressive symptoms can be observed in CEE in contrast to Scandinavia, where no significant association was observed, when all sociodemographic and health-related factors were accounted for. Northern European countries, viewed as nations with high welfare standards, good living conditions, and egalitarian society [42], employ preventive measures for mental health across many sectors, so EA alone is less impactful than in CEE, where such measures are lacking. We also found that EA poses considerably higher protection on mental health in adults younger than 65 years old. These results do not support the findings of Chevalier and Feinstein [43] where the positive effects of EA on mental health were observed at all ages or Bjelland [20] who argued that the benefits of EA accumulate throughout life. An explanation of our findings could be that developments in the quality of education over time have led to increasingly more effective and efficient activation of associated health promoting mechanisms such as improved socioeconomic status and healthy lifestyles across the life course. This would imply that younger populations may experience earlier and stronger protection against mental health problems than older ages. An alternative explanation is that mental health of older adults is affected by factors particularly related to aging, such as somatic comorbidities and physical functioning [2], which override the effects of EA.

Surprisingly, our study suggests that men in CEE, but not in other regions, benefit more from EA than women, while high EA seems unrelated to depressive symptoms in women. This phenomenon can be explained by the theory of resource multiplication by Ross and Mirowsky [44] stating that EA improves well-being more for men because the market payoffs they receive are greater. As men have more power, authority, and earnings, in addition to access to education, if these resources multiply each other’s impact, men could gain greater benefits from higher level of EA than women. Since women in CEE have traditionally faced worse opportunities than men [45] and have held primarily traditional caregiving roles in families and communities, the potential to capitalize and benefit from any EA is largely unrealized in this context. Ross and Mirowsky also hypothesized that the sex differences in depression may largely attenuate or even vanish as populations reach equality in EA [44], which supports our finding of no significant difference between men and women in more egalitarian societies.

An important consideration is the bidirectional nature of the association between EA and depressive symptoms. Individuals with predispositions to depression may not have pursued or succeeded in EA due to a range of mediators and associated factors having roots in depressive symptomatology. In a Canadian study, adolescents with depressive symptoms showed an increased risk of dropping out of high school when compared to their peers without depression [46]. In a US sample, depressive symptomatology was associated with increased odds of failure to complete high school only for girls, and among high school graduates, depressive symptoms were associated with failure to enter college in both men and women [47]. The discrepancies found in regards to EA for women with depressive symptoms in comparison to men highlight potential differences in the mechanisms which interact between EA, depression, and sex, and these associations warrant further investigation [48].

This study has several limitations. The cross-sectional observational design limits the interpretation of our results in terms of causality and has potential residual confounding. It is also not able to account for the longitudinal change of the EA between generations, which was undoubtedly prominent in 20th-century Europe. Our study sample is not entirely representative of adults older than 50 years as participants in SHARE are healthier and more educated than the general population. Additionally, we were unable to account for the time that EA was undertaken, therefore further exploration of the bidirectional relationship between EA and depression across the life course is warranted.

In the context of current demographic aging and increasing burden of mental disorders in Europe, this study is evidence toward the need to identify high-risk individuals for depression who would benefit from targeted preventive interventions. Increasing educational support, by introducing educational advisors, addressing the needs of low achievers or reducing the number of students in the classroom could serve as instructive examples of how EA could be modified. However, our study also supports the idea of limited mental health benefits in highly educated people, which should be explored more thoroughly in future research. These findings provide strong rationale and direction for public health strategies in Europe to more effectively target at-risk populations in lowering the burden of mental disorders in Europe.

## Data Availability

Access to the SHARE data is provided free of charge on the basis of a release policy that gives quick and convenient access to all scientific users worldwide after individual registration. All details about the application and registration process can be found on this website: http://www.share-project.org. The study protocol and syntax of the statistical analysis will be shared upon request from the corresponding author of this study.

## Supplementary material

For supplementary material accompanying this paper visit https://doi.org/10.1192/j.eurpsy.2020.100. click here to view supplementary material
