# Supplementary material for: Educational attainment inequalities in depressive symptoms in more than 100,000 individuals in Europe
Source: Eur Psychiatry. 2020 Nov 16;63(1):e97. doi: 10.1192/j.eurpsy.2020.100 (PMC7737177; doi:10.1192/j.eurpsy.2020.100)
Supplement: Supplementary file 1 [file S0924933820001005sup001.docx]

**Supplemental Table S.1** Characteristics of participants in different educational attainment levels

| EA | Level 0 (n=4876) | Level 1 (n=21805) | Level 2 (n=20932) | Level 3 (n=34747) | Level 4 (n=4340) | Level 5 (n=21103) | Level 6 (n=812) |
| --- | --- | --- | --- | --- | --- | --- | --- |
| Age, median (IQR) | 72 (16) | 68 (16) | 63 (15) | 61 (13) | 61 (14) | 60 (14) | 62 (15) |
| Women, n (%) | 2933 (60%) | 13027 (60%) | 12111 (59%) | 17918 (52%) | 2355 (54%) | 10350 (49%) | 299 (37%) |
| Years of education, median (IQR) | 3 (6) | 6 (3) | 9 (2) | 12 (2) | 13 (3) | 16 (3) | 19 (3) |

IQR, interquartile range

**Supplemental Table S.2** Association of educational attainment levels with depressive symptoms in the whole analytical sample comparing EURO-D scale cut-off of 4 and 7 points

| EA | Model 3_cut-off_4 | Model 3_cut-off_7 |
| --- | --- | --- |
| Level 0 | *Reference* | *Reference* |
| Level 1 | 0.79 (0.73; 0.85)** | 0.70 (0.63; 0.78)** |
| Level 2 | 0.71 (0.66; 0.77)** | 0.59 (0.52; 0.66)** |
| Level 3 | 0.67 (0.62; 0.72)** | 0.50 (0.44; 0.56)** |
| Level 4 | 0.68 (0.61; 0.75)** | 0.43 (0.35; 0.52)** |
| Level 5 | 0.60 (0.56; 0.66)** | 0.39 (0.34; 0.44)** |
| Level 6 | 0.76 (0.62; 0.92)* | 0.49 (0.30; 0.76)* |
| *AIC* | *101877* | 38444 |

Results are odds ratio with 95% confidence intervals derived from logistic regression for the association of educational attainment with depressive symptoms. The results are adjusted for age, sex, household net worth, employment status, family status, number of children, number of grandchildren, limitations in instrumental activities of daily living, 10 words delayed recall test, number of chronic diseases, chronic vascular disease, body mass index, physical activity, smoking, excessive alcohol intake, drugs for anxiety/depression, maximal grip strength

** p < 0.001; * p<0.05

AIC, Akaike information criterion


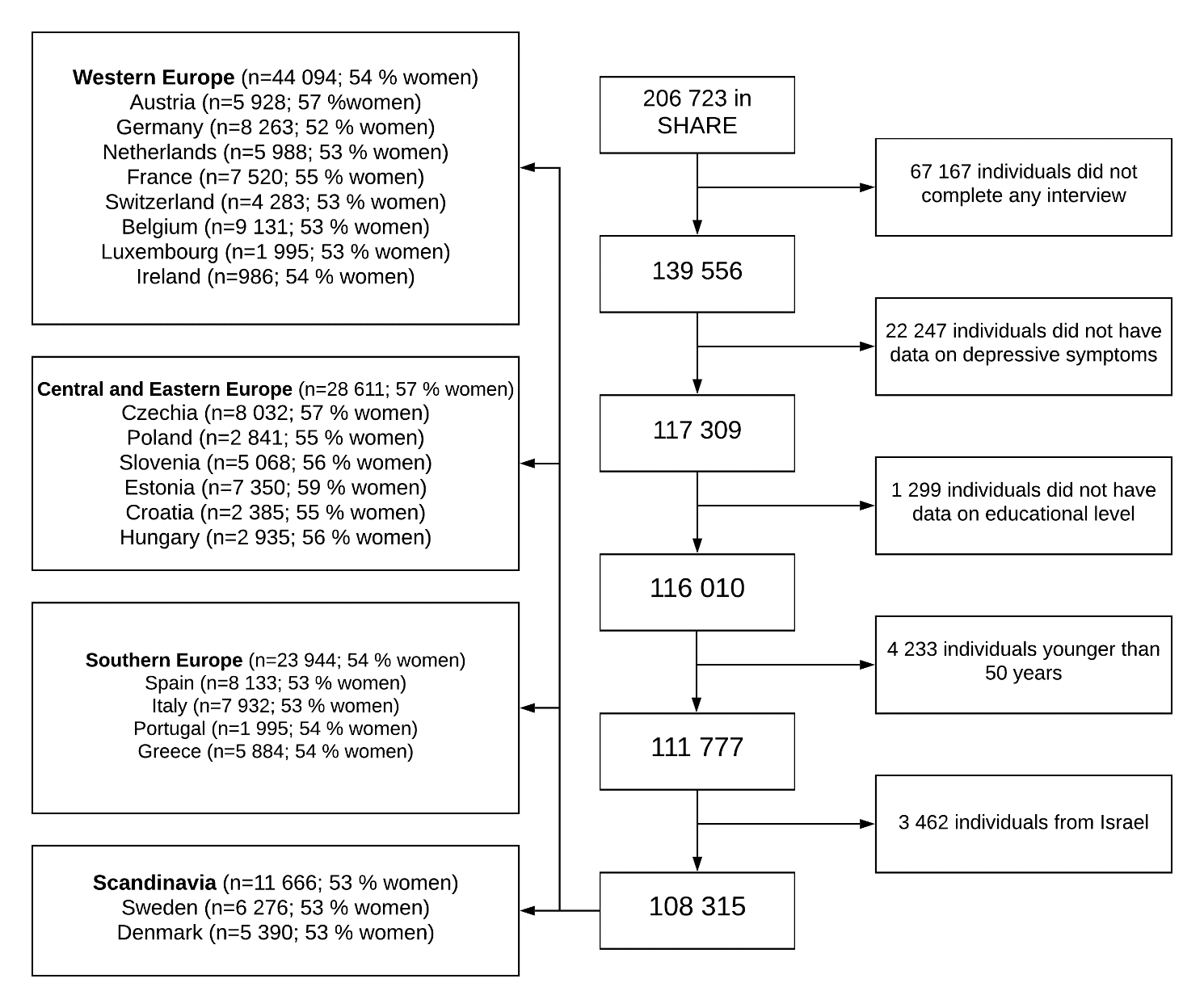


**Supplemental Figure S.1** Selection of the study sample
